# Asiatic Cholera; Its History, Causes and Prevention

**Published:** 1873-08

**Authors:** H. A. Buck

**Affiliations:** St. Louis, Mo.


*Asiatic Cholera; its History, Causes and Prevention.* By H. A. Buck, M.D., St. Louis, Mo.

We have very good authority for believing that epidemic cholera had its origin in the "*Black Death*" or "*Oriental Plague*" of the 14th century. And cholera, unquestionably, in all of its various forms, has destroyed more lives then all of the other epidemics taken together.

We are informed by historians that it swept off one-fourth of the inhabitants of the Old World, in the short period of four years.

England lost one-half of its population—London lost 100,000 in four months, and other cities in proportion.

It was preceded by great revolutions in the earth. From China to the shores of the Atlantic, the foundations of the earth were shaken, the atmosphere was in continual commotion; and endangered, by its poisonous influences, both vegetable and animal life. These terrible convulsions are said to have begun in 1333, fifteen years before the *plague* broke out in China.

According to the traditions of the Chinese, the pestilence was followed by torrents of rain, and great floods, and more than 400,000 people perished in consequence of the floods. A few months afterwards an earthquake caused an extensive range of mountains to sink and disappear, and in its place a lake was formed, more than 100 leagues in circumference—where thousands found a watery grave.

It is estimated that 4,000,000 people perished in China in the year 1337.

Europe was also visited with earthquakes and other atmospheric phenomena. These convulsions continued till 1347, when the plague or cholera, in its primitive form, broke out in the East.

This epidemic entered the western countries of Asia from China, in the year 1347, and here the historian obtains the first certain knowledge of the character of the disease. From China the route of commerce ran to the north of the Caspian Sea through Central Asia—from thence to Constantinople, the medium of communication between Asia, Europe and Africa. Thus in all directions contagion made its way, and doubtless Constantinople and the harbors of Asia Minor were the great centres of infection, whence it spread all over the world. People were struck down by *Black Death* as if by lightning, and the young and the strong were more frequently its victims than the aged and infirm. The descriptions of the disease given by historians, are rather indifferent. They inform us that sometimes it commenced with bleeding at the nose, which was a sure sign of death. Both in men and women, tumors in the groin, and inside of the thigh appeared at the beginning. These varied in size, but were frequently as large as an egg. Similar tumors appeared afterward all over the body, and black and blue spots came out on the arms and thighs.

These spots were indications of the fatal termination of the disease. No power of medicine brought relief; almost all died in from one to three days, and generally without fever or other symptoms—such was the form that the *Black Death* assumed in 1347, and three years following. In 1360 and 1373, it assumed a milder form, and then it was called the *Oriental Plague.* The tumors no longer appeared, and bleeding at the nose was seldom known to occur.

It has been supposed by excellent medical authority, that the form thus assumed by the *Oriental Plague*, is identical with the cholera of the present age, and that the term *Black Death* applied to it, was derived from the livid and frequently spotted appearance of patients in cases of malignant cholera. The most reliable estimates given, state that China lost 13,000,000 by *Black Death* or *Oriental Plague.*

India was almost depopulated, and Tartary and Syria, and all the adjoining countries, were literally covered with dead bodies.

Cyprus, it is said, lost all of its inhabitants; and ships without crews were seen, long after, floating about in the Mediterranean. It was reported that in the East, excepting China, 23,000,000 fell victims to this pestilence.

No climate was a barrier to its devastating and death-dealing power, it penetrated the icy regions of Greenland, Iceland, Norway and Denmark.

It reached the British Isles in 1349, and Russia in 1351, and according to all accounts it was not as fatal in the cold climates as it was in the hot climates. Thus we have the form and character of what is supposed to have been epidemic cholera in its primitive state.

Quite a period of time elapses before we have any description of this epidemic again, although it is supposed by some to have prevailed in a milder form in those countries where it first made its appearance.

It visited London again in the year 1666, and the mortality averaged one-fourth part of the population. During the cholera of 1832, the worst plague that has visited London since 1666, the deaths were only one out of two hundred and fifty inhabitants. This decrease of mortality was owing to the sanitary improvements in regard to cleanliness.

The next description that we have of it, is by a physician attached to the Dutch East India Company. His book was published in Batavia, in 1629. In describing what he calls epidemic cholera morbus, he says: "It is a disease of the most acute kind, and therefore requires immediate application. The animal spirits are speedily exhausted, and the heart, the foundation of life, is overwhelmed with putrid effluvia. Those who are seized with this disease generally die, and that so quickly as in the space of twenty-four hours at most. This disease is attended with a weak pulse, difficult respiration, and coldness of the extremities, to which are joined great internal heat, insatiable thirst, perpetual watching, and restlessness and incessant tossing of the body. If, together with these symptoms, a cold and fetid sweat should break forth, it is certain death." This I consider a very good description of the epidemic cholera of the present age.

During the latter part of the 17th century, and until the year 1774, the cholera appeared to have confined its ravages almost exclusively to the Hindoos.

Hindoo practitioners appear to have been familiar with its symptoms, or at least a disease closely resembling it, which they called *vishuchi*, a term in their language signifying vomiting and purging. In the first campaigns of the British troops in India, in the year 1774, cholera presenting all the symptoms known to characterize the epidemic, made its appearance at Madras, and proved very fatal to both the European and native soldiers, carrying them off very quickly after they were attacked. It is asserted that over 60,000 people perished between the years 1774 and 1781. The disease prevailed at various times between the years 1783 and 1790, and always with the same symptoms and with the same fatal results.

In the year 1783, the cholera broke out at the sacred bathing place on the Ganges. This was the year of the great pilgrimage; it is said 2,000,000 were assembled at this time. In eight days 20,000 fell victims. It did not extend beyond the place of bathing, and ceased on the dispersion of the multitude. In 1817, it broke out again on the banks of the Ganges, causing a greater destruction of life than at any other time.

By the end of 1818 its ravages embraced nearly all of Hindostan, and in 1819 it appeared in Java, in the Isles of France and Bourbon, and over India and China; in 1821, in Bagdad, Arabia; and in 1822, in Persia and Syria. In 1823 it broke out in Antioch, Tripoli, and all along the Mediterranean coast. In 1823 and 1827, the disease continued its ravages in China and India—in 1828 the Russian Empire was again invaded. The cold of the preceding winter stayed its progress, but the summer of 1829 found it committing terrible havoc in the same localities.

In 1830 it appeared in the Georgian cities, and in Poland. In 1831 in Warsaw, St. Petersburg, and all the principal cities of Russia, Prussia, Austria and Italy; and the same year it made its appearance in England, Scotland and Ireland. In 1832, in the month of March, it appeared in Dublin, and in May, in Paris.

Its first appearance in the New World, was in June, 1832, at Montreal and Quebec, and during its prevalence the mortality was great.

Since its appearance on our shores it has been almost periodical, visiting a place in its epidemic form once in sixteen or seventeen years, and remaining from one to three years, during the hot months of the year, if it found a soil congenial for its germs to propagate in.

Epidemic cholera has appeared in St. Louis twice* at an interval of about sixteen years. On its appearance in 1849, there were some 65,000 inhabitants in St. Louis. It is thought some 15,000 left the city; the statistics give the mortality at 6,000.

Let us compare the mortality of St. Louis with Boston and Baltimore, where strict sanitary laws were enforced in the same years. Boston appropriated a sum of money sufficient to cleanse and purify the streets, alleys and yards. The work was done thoroughly and effectively.

* *Three times.*—First in 1833 and 1834. We have little more faith in the sixteen or seventeen-year periodicity of cholera than we have in the "forty-year flood of the Mississippi."—ED. ST. LOUIS M. & S. JOUR.

In St. Louis no attempt was made to remove the filth, or in any way to improve the sanitary condition of the city, except what may have been done by a few individuals on their own premises.

Now compare the results. Boston, out of a population of 140,000, lost but 327 by cholera, from all causes, 5,000—while in St. Louis, with less than 65,000 inhabitants, 6,000 persons died of cholera alone.

In Baltimore $40,000 was appropriated by the city council, for the work of purification, in anticipation of the coming epidemic. The result was, that out of a population of 160,000, but 853 died of cholera. Showing, I think, conclusively, that cholera can be prevented from appearing in an epidemic form.

I was in New York when epidemic cholera prevailed in 1850, 1851 and 1852. It was the last of September when the cholera broke out in an orphan asylum. The Superintendent took the orphans out on a picnic, and immediately on their return the cholera attacked them in a very malignant form. The Superintendent and some thirty out of ninety cases died. I think the great mortality in the asylum was in consequence of worthless nurses (as they had to depend on the alms-house for nurses on that occasion), badly ventilated rooms, and not separating the sick from the well. I was a student of medicine, and helped to take care of them, and formed a very unfavorable opinion of the curative properties of medicine in epidemic cholera at that time. In 1866, when the cholera attacked the inmates in any of the public institutions and hospitals in New York, they removed them to wards by themselves, and they disinfected the wards, and the consequence was, there were very few cases compared with former epidemics. I was one of the Ward physicians of the Sixth Ward, in St. Louis, in 1866. Our Ward extended from the levee to the city limits west.

The cholera that year was confined mostly to the crowded tenement buildings on the principal streets and alleys. There were a few cases in the western portion of the city, where the buildings stood isolated, and it was thought by some, it was in consequence of drinking the well water in those localities that were subject to surface drainage.

Its second appearance in an epidemic form in St. Louis (as you all know) was in 1866. I have not the statistics of the number of cases and deaths at its last visitation in St. Louis, but there were not as many, according to the number of inhabitants, as in 1849. But I believe we had more cases than there were in New York in the same years, 1866 and 1867.

According to the statistics of the Board of Health for New York and Brooklyn, they only had 1,909 cases of cholera in the year 1866; they do not give the number of deaths for that year. In 1867 they only had twenty-seven deaths from cholera. And I

think it can be attributed to the great vigilance of the Board of Health of New York, that it was prevented from making such ravages as in its former visitations in that city. Now, unless there is a new departure in the periodicity of epidemic cholera, we need not fear another visitation of it again until 1882 or 1883, and not then if there is a strict observance of sanitary laws and hygiene.

We stated in the former part of this paper, that there were great floods, converting immense tracts of land into swamps. And as the water subsided, the flooded districts were drained, foul vapors arose everywhere, from decomposing vegetable and animal matter, made more horrible and poisonous by the odor of putrefied corpses, the victims of floods, famines and earthquakes.

It is probable that the atmosphere thus contaminated, gave origin to the *Black Death*, whenever the organs of respiration came in contact with it. At a later period it was stated that its origin in several instances, was traced to the pilgrims, who visited the banks of the Ganges, in the observance of their religious rites.

And still later, the havoc committed by the epidemic at Alexandria, suggested official inquiries, and the President of the Board of Health addressed a communication on the subject to the Minister of Foreign Affairs. In this paper it is stated as the opinion, not only of the President himself, but of all the scientific and professional authorities in Egypt, that the poison is generated by crowds of pilgrims periodically visiting the holy places of Arabia. The pilgrims congregate at certain periods of the year, from all parts of the Mohamedan World, to the number of seven or eight hundred thousand.

It is ever a point of religion with them that no pilgrim should change his clothes during the whole time of his pilgrimage. Under these conditions they are huddled together in enormous crowds beneath the fiery sky of the desert. It is an indispensable incident of the ritual that each pilgrim should sacrifice at least one sheep, and the skin and offal of these countless victims are left to decompose under an Arabian sun. The result of all this is that thousands of pilgrims perish on the spot, leaving their bodies to be shuffled hastily under a coating of sand which the first sirocco will disperse; and their clothes to be packed up and carried off as relics to be distributed among their relations and countrymen. The Egyptian minister thinks that here is the seed-plot and hot-bed of cholera.

It will scarcely be questioned that epidemic cholera is a disease originating in a specific principle pervading the atmosphere; this, acting in combination with miasmata rising from the earth, creates a positive poison, and this is productive of the same results wherever it comes in contact with the vital organs.

But while it produces but one disease there are distinct stages of the same, the different stages, in some cases, being more or less merged into one; but in nearly every instance the peculiarities of each are plainly observable. How this atmospheric poison originates, or in what manner it attacks the system, we can only venture to express our opinion. It may act indirectly through the nervous system, thence affecting the organs of sympathy and sensation. Some distinguished medical gentlemen contend that it enters through the medium of the lungs. The discussion of the subject must be, after all, a mere matter of speculation, as it is not known how it enters the system. Neither is the nature of the cholera-poison known. The fact, however, is well known that a malarious poison does exist, and that, combined with local causes, it operates with deadly power. By imprudence in living, fatigue, want of sleep, gorging the stomach with food, stimulating the brain with alcoholic drinks, giving way to fear, or, in fact, any irregularity having a tendency to derange the course of nature, the magazine is prepared with explosive material, only waiting the epidemic match to hurl its victims to destruction.

How shall we prevent epidemic cholera? In reference to this subject the first thing to be considered is the fact that cholera is dependent primarily on atmospheric conditions, and proximately on local conditions, impure air being the most conspicuous. The mysterious principle, whatever it may be, generated in the great crucible of nature—whether in the jungles of India, the simoons of Arabia, or the craters of Vesuvius—may be carried on the wings of the wind over the surface of the earth, without producing more than a slight disturbance of the functions of animal life, manifested in a tendency to disease of the bowels, and general depression of the vital powers. Another poisonous principle is generated in the laboratories of terrestrial filth. Every city and town sends up its deleterious gases, its disease-laden malaria, which combines with the poisonous principle with which the atmosphere is already impregnated, and thus a compound is generated, which may be the fatal cholera-poison.

Predisposition and susceptibility favor the operation of the prevailing cause, therefore sanitary measures should be promptly instituted for the thorough purification of these gas-generating foci all around us in full blast; from the offal in vacant lots; from the alleys reeking with pollution; from the gutters and back yards of tenement houses.

This is where sanitary labors should begin. Competent sanitarians in every community, great or small, should inspect this work.

Timely and thorough *cleansing* and *disinfecting* is what "stamp out" Asiatic or sporadic cholera, better than any other means yet known to civilization.—*St. Louis Med. and Surg. Journal.*

